# *Anopheles farauti* is a homogeneous population that blood feeds early and outdoors in the Solomon Islands

**DOI:** 10.1186/s12936-016-1194-9

**Published:** 2016-03-09

**Authors:** Tanya L. Russell, Nigel W. Beebe, Hugo Bugoro, Allan Apairamo, Frank H. Collins, Robert D. Cooper, Neil F. Lobo, Thomas R. Burkot

**Affiliations:** Australian Institute of Tropical Health and Medicine, James Cook University, Cairns, QLD 4870 Australia; School of Biological Sciences, University of Queensland, St. Lucia, QLD 4068 Australia; CSIRO, Dutton Park, Brisbane, QLD 4102 Australia; National Vector Borne Disease Control Programme, Ministry of Health, Honiara, Solomon Islands; Department of Biological Sciences, Eck Institute for Global Health, University of Notre Dame, Notre Dame, IN 46556 USA; Australian Army Malaria Institute, Gallipoli Barracks, Enoggera, 4052 Australia

**Keywords:** Behavioural polymorphism, *Anophelesfarauti*, Solomon Islands, Mark-release-recapture, Heterogeneous population, Insect

## Abstract

**Background:**

In the 1970s, *Anopheles farauti* in the Solomon Island responded to indoor residual spraying with DDT by increasingly feeding more outdoors and earlier in the evening. Although long-lasting insecticidal nets (LLINs) are now the primary malaria vector control intervention in the Solomon Islands, only a small proportion of *An. farauti* still seek blood meals indoors and late at night where they are vulnerable to being killed by contract with the insecticides in LLINs. The effectiveness of LLINs and indoor residual spraying (IRS) in controlling malaria transmission where the vectors are exophagic and early biting will depend on whether the predominant outdoor or early biting phenotypes are associated with a subpopulation of the vectors present.

**Methods:**

Mark-release-recapture experiments were conducted in the Solomon Islands to determine if individual *An. farauti* repeat the same behaviours over successive feeding cycles. The two behavioural phenotypes examined were those on which the WHO recommended malaria vector control strategies, LLINs and IRS, depend: indoor and late night biting.

**Results:**

Evidence was found for *An. farauti* being a single population regarding time (early evening or late night) and location (indoor or outdoor) of blood feeding. Individual *An. farauti* did not consistently repeat behavioural phenotypes expressed for blood feeding (e.g., while most mosquitoes that fed early and outdoors, and would repeat those behaviours, some fed late at night or indoors in the next feeding cycle).

**Conclusions:**

The finding that *An. farauti* is a homogeneous population is significant, because during the multiple feeding cycles required to complete the extrinsic incubation period, many individual female anophelines will enter houses late at night and be exposed to the insecticides used in LLINs or IRS. This explains, in part, the control that LLINs and IRS have exerted against a predominantly outdoor feeding vector, such as *An. farauti*. These findings may be relevant to many of the outdoor feeding vectors that dominate transmission in much of the malaria endemic world and justifies continued use of LLINs. However, the population-level tendency of mosquitoes to feed outdoors and early in the evening does require complementary interventions to accelerate malaria control towards elimination.

## Background

Environmental conditions outside of that normally experienced by a population are closely associated with phenotypic change in that population [1, 2], and one common example of a changed environment is prolonged exposure of insects to insecticides. The response of an insect population to insecticide pressure is governed by interacting ecological phenomena and depends on numerous factors including the timing and magnitude of pressure as well as the life-cycle stage that is targeted. In response to ecological change, populations may respond with allee effects, viability selection, fecundity selection, maternal effects and within-individual phenotypic plasticity [3]. Understanding these responses is essential for defining policy and optimising vector control operations [4].

Anopheline mosquito populations were under intensive pressure from extensive DDT applications during the Global Malaria Eradication Program (GMEP), launched in 1955, and one of the technical reasons given for the failure to eliminate malaria was behavioural adaptations by the mosquito vectors [5–7]. Those mosquito populations that responded by changing their behaviour to avoid DDT by feeding and/or resting outdoors had a selective advantage. This was observed after DDT use in various species including *Anopheles farauti* [8, 9], *An. sundaicus* [10]*, An. pseudopunctipennis* [11, 12] and *An. albimanus* [11–13]. Towards the end of the GMEP, transmission continued in many “problem areas” by physiologically susceptible vectors that avoided or minimized their exposure to DDT [5–7]. However, the mechanistic drivers of behavioural resistance remain undefined. One possible explanation is that specific karyotype inversions associated with feeding or resting outdoors became assimilated in the population through selection.

Another unresolved question is whether insecticidal pressure was uniform and led to behavioural changes across entire mosquito populations or if subpopulations developed with different behaviours. The existence of subpopulations with associated behavioural phenotypes could further threaten the impact of malaria vector control measures if the subpopulation has an associated phenotype rendering it less susceptible to a vector control intervention; for instance exclusive exophagy (outdoor feeding) would make bednets inconsequential. The hypothesis that subpopulations may be evolving is drawn from observations in West Africa, where *An. gambiae* sensu stricto displays intraspecific polymorphism with sympatric taxa having characteristic combinations of chromosomal inversions (Mopti, Bamako, Savanna, Bissau and Forest) [14]. These distinct subpopulations display different ecological tolerances [14, 15]. For example, the Mopti form has the unique ability to breed throughout the dry season in irrigated areas [16]. For *An. arabiensis* (a member of the *An. gambiae* complex), simultaneous collections of *An. arabiensis* made in Nigeria from inside and outside of houses had associated inversion karyotypes [17].

In Melanesia, isolated island populations of *An. farauti* vary in their night biting profile and degree of endophily [18–20], and this variance is likely to have resulted from restricted gene flow between islands [21]. The behavioural phenotypes that directly determine the effectiveness of indoor vector control with long-lasting insecticidal nets (LLINs) and indoor residual spraying (IRS) are the time (nocturnality) and location (endophagy) of blood feeding. A series of mark-release-recapture experiments were undertaken to define the consistency of these key behavioural phenotypes in an *An. farauti* population.

## Methods

### Study site

The study was conducted in Haleta village on Ngella Sule Island in Central Province, Solomon Islands (−9°5′56″S, 160°6′56″E) [23]. This rural coastal village of 470 people is bounded by the ocean to the south and rugged mountains to the north (Bed net census, 2010, Solomon Islands Ministry of Health, unpublished data). The climate is hot and wet and has an annual rainfall of 2837 mm (based on 43 years of data collected at the provincial capital Tulagi approximately 10 km from Haleta village) [24]. The mean annual temperature is 26 °C with daily temperatures ranging from 30 °C during the day to 24 °C at night and is reasonably constant throughout the year. Malaria is vectored by *An. farauti* sensu stricto.

### Mosquito sampling and processing

The mark-release-recapture experiments sampled *An. farauti* by human landing catch (HLC) [25] over 14 consecutive nights. For the initial 7 nights of each experiment, wild blood-fed female *An. farauti* of unknown chronological age were marked with fluorescent dust and released. For nights 2 through 14, all captured *Anopheles* were visually checked for dust markings using a UV torch and any recaptured marked mosquitoes were separated prior to further manipulations. HLC stations were located along the length of the village [23]. Captured mosquitoes were held in individual waxed paper cups for each hour and location of collection. Each hour, all *Anopheles* were morphologically identified [26] and counted by hour and location of collection.

**Fig. 1:**
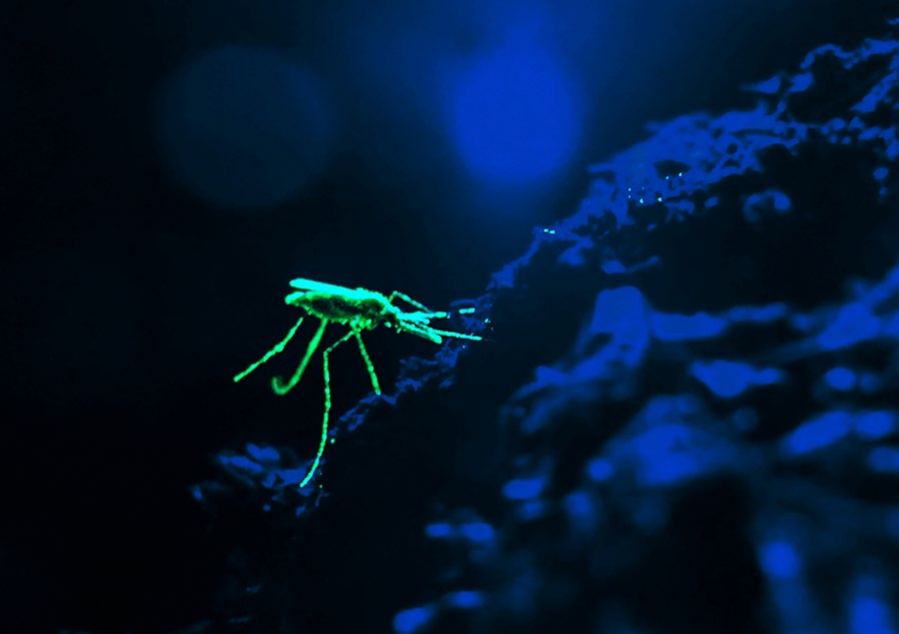
*Anopheles* *farauti* mosquito that has been marked with green fluorescent dust for the mark-release-recapture experiment

Captured *An. farauti* were pooled by time of collection (e.g. early [18.00–21.00 h] or late feeding [00.00–06.00 h] or location of collection [e.g., indoors or outdoors]). These *An. farauti* were marked with a unique fluorescent dust colour for time or location of collection (Fig. 1). For marking, a maximum of 100 blood-fed mosquitoes were placed into plastic 250 ml cups covered with netting. A small amount of fluorescent powder (BioQuip Products, Inc. California, USA and Glow Paint Industries, Queensland, Australia) was sifted through the netting into the cup; a fine tipped transfer pipette was used to aerosolize the powder which coated the mosquitoes. The effectiveness of this procedure was checked by examining the mosquitoes in each cup with a LED UV torch (400 nm wavelength) to ensure that they were adequately marked with the powder. The mosquitoes were released on the night of collection from a single outdoor location. The distance from the release site to the furthest HLC collection station was 190 m. All mosquitoes not released were stored in 100 % ethanol in micro-centrifuge tubes. A sample of the captured *An. farauti* was identified by molecular analysis of the internal transcribed spacer region II of the ribosomal DNA [27].

### Experiment 1: Time of host-seeking: early versus late feeding

The hypothesis of experiment 1 tested if there were separate early and late feeding populations of *An. farauti* (e.g., do individual *An. farauti* repeatedly feed at the same time of the night or do individual *An. farauti* sometimes feed early and other times feed late?). The experiment was conducted from 23rd November–6th December 2011. Mosquitoes were sampled from 6 outdoor collection stations from 18.00–06.00 h. Mosquito collectors worked from 18.00–00.00 h and were replaced by a second team that worked from 00.00–06.00 h. Early host-seeking mosquitoes were defined as those that were captured by HLC between 18.00–21.00 h; late night host-seeking mosquitoes were defined as those captured by HLC between 00.00–06.00 h. A 3 h period (21.00–00.00 h) provided a buffer between the early and late phenotypes. The mosquitoes caught early and those captured late were marked with different colour fluorescent dusts before release. Marked blood-fed *An. farauti* were released shortly after 06.00 h. The *An. farauti* specimens were processed as described above in “Mosquito sampling and processing” section.

### Experiment 2: Host-seeking location: indoor versus outdoor feeding

Experiment 2 tested the hypothesis of whether separate indoor and outdoor feeding populations of *An. farauti* exist [e.g., do individual *An. farauti* repeatedly seek blood meals at the same location (indoors or outdoors) in consecutive feeds]. The experiment was conducted over 14 consecutive nights from 2 to 16 May 2012. *Anopheles farauti* were sampled from 10 collection stations by HLC between 18.00–00.00 h. Each sampling station consisting of two collection sites: one site was inside a house and the second site was outdoors at a distance of at least 10 m from the indoor collector. The *An. farauti* caught indoors or outdoors were marked with different colours of florescent dust to differentiate the location (indoors or outdoors) at which they were initially caught host-seeking. Marked *An. farauti* were released shortly after midnight. The *An. farauti* specimens were processed as described above in “Mosquito sampling and processing” section.

### Statistical analysis

The data was compiled in a series of tables which detailed the results of: (1) mosquito collections, (2) molecular analyses and (3) mark-release-recapture releases [28]. The results of the mark-release-recapture experiments were analysed using generalized linear models (GLMs) with a binomial distribution and a categorical explanatory variable for mosquito label (i.e. unmarked or dusted). For experiment 1 (time of host seeking), the dependent binary variable contained the number of mosquitoes caught biting either early or late in the night. For experiment 2 (host-seeking location), the dependent binary variable contained the number of mosquitoes caught biting either indoors or outdoors. All analyses were conducted using the *R* package V3.1.2 [29].

### Ethics

Ethical approval for the study was obtained from the National Health Research and Ethics Committee, Solomon Islands (02-05-2011), the James Cook University Human Research Ethics Committee, Australia (H4122) and the University Hospitals Case Medical Centre Institutional Review Board for Human Investigation, USA (05-11-11). Mosquito collectors were recruited from the village residents after the risks were explained and they signed an informed consent agreement. Only village adults who likely have some immunity to malaria were asked to participate in the landing catches and were instructed to capture the mosquitoes before they bite, and all took malaria prophylaxis. To estimate the duration of the feeding cycle by mark-release-recapture, mosquitoes were offered a human blood meal prior to release from authors on malaria prophylaxis.

## Results

### Experiment 1: Time of host seeking: early versus late feeding

**Fig. 2:**
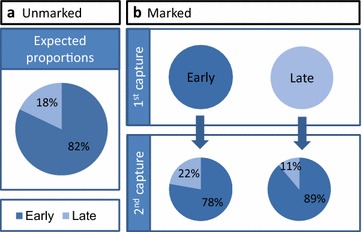
The preference of *An. farauti* to host-seek early (18.00–21.00 h) or late (00.00–06.00 h) as examined by a mark-release-recapture experiment. The expected proportion was calculated directly for the overall human landing catch dataset (**a**). The second capture indicates where mosquitoes were recaptured after they were marked and released (**b**)

Over the 14 night experiment, 1388 *An. farauti* were caught by HLC. A sample of the specimens (n = 92) were confirmed as *An. farauti s.s.* by molecular analysis. There was a strong tendency for mosquitoes to feed early in the evening, with 82 % (n = 934) of *An. farauti* caught between 18.00 and 21.00 h (i.e. early feeding), and only 18 % (n = 204) of *An. farauti* caught between 00.00 and 06.00 h [i.e. late feeding; noting that this ratio excluded the portion of mosquitoes (n = 248) caught between 21.00 and 00.00 h]. A total of 618 marked *An. farauti* were released (510 initially caught early and 108 initially caught late). The overall recapture rate of marked *An. farauti* was 3.6 % (n = 18). Of the *An. farauti* that were released after being captured early, 78 % (n = 7) were recaptured during this same time (18.00–21.00 h), with 22 % (n = 2) being recaptured late at night (between 00.00–06.00 h). Of the mosquitoes that were originally captured after midnight, 89 % (n = 8) were sequentially recaptured feeding early (18.00–21.00 h) and only 11 % (n = 1) was recaptured during the late night feeding period (00.00–06.00 h; Fig. 2). The ratio of early to late biting mosquitoes was not significantly different between the unmarked population and either of the recaptured populations (Table 1).

**Table 1:** A comparison of the proportion of mosquitoes caught biting either early or late in the night for each experimental mosquito label and analysed with a binomial generalized linear model (GLM)

Label of captured mosquitoes	Proportion caught early (n/total)	Odds ratio (se)	*p* value
Unmarked	0.82 (911/1112)		
Released from early	0.78 (7/9)	0.773 (0.805)	0.748
Released from late	0.89 (8/9)	1.765 (1.063)	0.593

### Experiment 2: Host-seeking location: indoor versus outdoor feeding

**Fig. 3:**
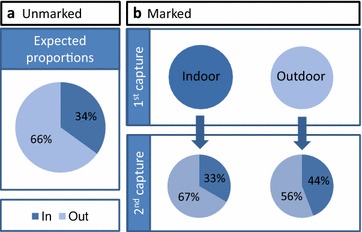
The preference of *An. farauti* to host-seek indoors or outdoors as examined by a mark-release-recapture experiment. The expected proportion was calculated directly for the overall human landing catch dataset (**a**). The second capture indicates where mosquitoes were recaptured after they were marked and released (**b**)

Over the 14 night experiment, 1008 *An. farauti* were caught by HLC. A sample of the specimens (n = 28) were confirmed as *An. farauti s.s.* by molecular analysis, with nine being recaptured specimens. Sixty-five percent (n = 654) of *An. farauti* were initially captured outdoors with 35 % (n = 354) captured indoors. A total of 684 *An. farauti* were marked with fluorescent dusts and released (482 initially caught outdoors and 202 initially caught indoors). The overall recapture rate of marked *An. farauti* was 13.0 % (n = 89). Of the *An. farauti* that were originally captured indoors, 67 % (n = 14) were recaptured outdoors with 33 % (n = 7) recaptured indoors. Of the *An. farauti* that were originally captured outdoors, 56 % (n = 38) were subsequently recaptured outdoors with 44 % (n = 30) recaptured indoors (Fig. 3). There was no significant difference in the ratio of indoor to outdoor biting *An. farauti* found between the unmarked and recaptured populations (Table 2).

**Table 2:** A comparison of the proportion of mosquitoes caught biting either indoors or outdoors for each experimental mosquito label and analysed with a binomial generalized linear model (GLM)

Label of captured mosquitoes	Proportion caught indoors (n/total)	Odds ratio (se)	*p* value
Unmarked	0.34 (317/919)		
Released from indoors	0.33 (7/21)	0.949 (0.468)	0.912
Released from outdoors	0.44 (30/68)	1.499 (0.254)	0.111

## Discussion

Evidence was found for *An. farauti* being a single population; although it usually feeds outdoors and early, it sometimes feeds indoors and late. This population of mosquitoes was studied because it had altered its preferred feeding behaviour after prolonged insecticide exposure (initially DDT-IRS, and then insecticide treated nets and/or pyrethroid-IRS) [22, 30, 31]. Recent studies of *An. farauti* populations in the Solomon Islands observed a strong tendency for females to bite outdoors and early in the evening. The reported proportion of blood meals on humans taking place indoors (π_i_ values) ranged from 0.130 to 0.546 across Central, Guadalcanal, Temotu and Isabel Provinces [18–20, 23].

Prior to *An. farauti* changing its feeding behaviour to what is expressed today, the populations were homogeneous for host seeking behaviour. Previously in Madang, Papua New Guinea and Guadalcanal, Solomon Islands, the possibility of subpopulations of indoor/outdoor or early/late feeding *An. farauti* was directly examined with mark-recapture experiments but no heterogeneity in the population was evident [32, 33]. At the time when these experiments were conducted in the 1980s, the *An. farauti* populations exhibited almost even indoor and outdoor biting in Madang and Guadalcanal [33, 34] with the Madang population expressing a nocturnal biting behaviour with peak biting in the middle of the night [34].

The degree to which the vector feeds indoors (endophagy) and during times when humans are sleeping (nocturnal) will largely determine the effectiveness of LLINs by determining the proportion of vectors that are potentially exposed to the insecticide during each feeding cycle. The finding that *An. farauti* is a homogeneous population in terms of feeding behaviour is significant as it means that the population is composed of individuals whose potential insecticide exposure to LLINs is uniform. Across multiple feeding cycles, each individual female can potentially enter houses and be exposed to the insecticide in LLINs. Hence, although *An. farauti* is primarily exophagic, indoor vector control tools still provide an important level of control [35]. Another anopheline population with homogenous indoor/outdoor biting feeding was *An. maculatus* in Malaysia [36]. However, significant heterogeneity in indoor/outdoor resting populations was found associated with polymorphic isozyme loci for *An. balabacensis* in Malaysia [37, 38].

Evidence has been emerging that other anopheline mosquito populations are altering their behaviour to avoid prolonged insecticide exposure, such as *An. funestus* in Tanzania [39], Benin [40] and Senegal [41] as well as *An. gambiae s.s.* in Equatorial Guinea [42]. The underlying mechanisms for the altered behaviour of *An. funestus* and *An. gambiae* are unknown [43]. Nonetheless, the risks of behavioural phenotype change to effective mosquito control with LLINs and IRS are uncertain but are likely to be variable depending on the proportions of vector populations that are endophagic and nocturnal and whether the vector population is comprised of subpopulations associated with endophagy and time of feeding.

## Conclusion

Evidence was found for a behaviourally homogeneous population of *An. farauti*. *Anopheles farauti* does not have subpopulations that consistently seek blood meals either indoors or outdoors or that seek blood meals early as opposed to late at night. This is significant because, while the probability of any individual female mosquito entering a house late at night to blood feed is low during any one feeding cycle, over the course of the extrinsic incubation period composed of multiple feeding cycles, the potential to be exposed to insecticides in LLINs (or IRS) is significant. Hence, even highly exophilic and early feeding vectors can be controlled by interventions that are implemented inside houses. However, malaria elimination is unlikely to be obtained by vector control in the absence of additional interventions that complement the indoor protection afforded by LLINs and IRS by killing or minimizing man-vector contract outside of houses.

## Availability of data and materials

The datasets supporting the conclusions of this article are available in the James Cook University Tropical Data Hub repository: http://dx.doi.org/10.4225/28/56BD124CC9260.

## Abbreviations

GLM: generalized linear model
GMEP: Global Malaria Eradication Program
HLC: human landing catch
IRS: indoor residual spraying
LLINs: long-lasting insecticidal nets

## References

[1] Badyaev AV (2005). Stress-induced variation in evolution: from behavioural plasticity to genetic assimilation. Proc R Soc Lond B.

[2] Hoffman AA, Hercus MJ (2000). Environmental stress as an evolutionary force. Bioscience.

[3] Candolin U, Wong BB (2012). Behavioural responses to a changing world: mechanisms and consequences.

[4] Ferguson H, Maire N, Takken W, Lyimo I, Briet O, Lindsay S (2012). Selection of mosquito life-histories: a hidden weapon against malaria?. Malar J.

[5] Elliott R (1972). The influence of vector behaviour on malaria transmission. Am J Trop Med Hyg.

[6] Hamon J, Mouchet J, Brengues J, Chauvet G (1970). Problems facing Anopheline vector control. Vector ecology and behaviour before, during, and after application of control measures. Misc Publ Entomol Soc Am.

[7] Mattingly PF (1962). Mosquito behaviour in relation to disease eradication programmes. Annu Rev Entomol.

[8] Slooff R. Observations on the effect of residual DDT house spraying on behaviour and mortality in species of the *Anopheles punctulatus* group. Final report on a research project in West New Guinea [PhD Thesis]. Leyden: University of Leyden; 1964.

[9] Thevasagayam ES (1983). Malaria control strategies in the Southwest Pacific countries-reappraisal.

[10] Sundararaman S (1958). The behaviour of *A. sundaicus* Rodenwaldt in relation to the application of residual insecticides in Tjilatjap, Indonesia. Indian J Malariol.

[11] de Zulueta J, Garrett-Jones C (1963). An investigation of the persistence of malaria transmission in Mexico.

[12] Martinez-Palomo A, de Zulueta J (1964). Ethological changes in *Anopheles pseudopunctipennis* in Mexico after prolonged use of DDT.

[13] Trapido H (1952). Modified response of *Anopheles albimanus* to DDT residual house spraying in Panama. Am J Trop Med Hyg.

[14] Krzywinski J, Besansky NJ (2003). Molecular systematics of *Anopheles*: from subgenera to subpopulations. Annu Rev Entomol.

[15] White BJ, Collins FH, Besansky NJ (2011). Evolution of *Anopheles gambiae* in relation to humans and malaria. Annu Rev Ecol Evol Syst.

[16] Gimonneau G, Bouyer J, Morand S, Besansky NJ, Diabate A, Simard F (2010). A behavioral mechanism underlying ecological divergence in the malaria mosquito *Anopheles gambiae*. Behavioral Ecol.

[17] Coluzzi M, Sabatini A, Petrarca V, Di Deco MA (1977). Behavioural divergences between mosquitoes with different inversion karyotypes in polymorphic populations of the *Anopheles gambiae* complex. Nature.

[18] Bugoro H, Cooper R, Butafa C, Iro’ofa C, Mackenzie D, Chen C-C (2011). Bionomics of the malaria vector *Anopheles farauti* in Temotu Province, Solomon Islands: issues for malaria elimination. Malar J.

[19] Bugoro H, Iro’ofa C, Mackenzie D, Apairamo A, Hevalao W, Corcoran S (2011). Changes in vector species composition and current vector biology and behaviour will favour malaria elimination in Santa Isabel Province, Solomon Islands. Malar J.

[20] Bugoro H, Hii J, Butafa C, Iroofa C, Apairamo A, Cooper R (2014). The bionomics of the malaria vector *Anopheles farauti* in Northern Guadalcanal, Solomon Islands: issues for successful vector control. Malar J.

[21] Ambrose L, Cooper RD, Russell TL, Burkot TR, Lobo NF, Collins FH (2014). Microsatellite and mitochondrial markers reveal strong gene flow barriers for *Anopheles farauti* in the Solomon Archipelago: implications for malaria vector control. Int J Parasitol.

[22] Russell TL, Beebe NW, Cooper RD, Lobo NF, Burkot TR (2013). Successful malaria elimination strategies require interventions that target changing vector behaviours. Malar J.

[23] Russell TL, Beebe NW, Bugoro H, Apairamo A, Chow W, Cooper RD et al. Frequent blood feeding enables insecticide-treated nets to reduce transmission by mosquitoes that bite predominately outdoors. Malar J. 2016. doi:10.1186/s12936-016-1195-8. 10.1186/s12936-016-1195-8 PMC4788858 26969430

[24] Brookfield HC, Hart D. Rainfall in the tropical southwest Pacific. Canberra: Department of Geography, Publ G/3, The Australian National University; 1966.

[25] Gimnig JE, Walker ED, Otieno P, Kosgei J, Olang G, Ombok M (2013). Incidence of malaria among mosquito collectors conducting human landing catches in Western Kenya. Am J Trop Med Hyg.

[26] Belkin JN (1962). The mosquitoes of the South Pacific (Diptera, Culicidae).

[27] Beebe NW, Saul A (1995). Discrimination of all members of the *Anopheles punctulatus* complex by polymerase chain reaction—restriction fragment length polymorphism analysis. Am J Trop Med Hyg.

[28] Russell TL, Beebe NW, Bugoro H, Apairamo A, Cooper RD, Lobo NF et al. Dataset for mark-release-recapture experiments detailing the place and time of feeding by Anopheles farauti in Haleta village, Solomon Islands. James Cook University Tropical Data Hub; 2016. doi:10.4225/28/56BD124CC9260.

[29] R Core Team. R: A language and environment for statistical computing. Vienna: R Foundation for Statistical Computing; 2013.

[30] Taylor B (1975). Changes in the feeding behaviour of a malaria vector, *Anopheles farauti* Lav., following the use of DDT as a residual spray in houses in the British Solomon Islands Protectorate. Trans R Entomol Soc London.

[31] Paik Y-H, Avery JG (1973). Problem areas in the malaria eradication programme in the British Solomon Islands. P N G Med J.

[32] Charlwood JD, Graves PM, Birley MH (1986). Capture-recapture studies with mosquitos of the group of *Anopheles punctulatus* Dönitz (Diptera, Culicidae) from Papua New Guinea. Bull Entomol Res.

[33] Hii JLK. Antimalaria program. WHO assignment report. (WP)MAL/SOL/MAL/001-E. Honiara: WHO Regional Office for the Western Pacific; 1988.

[34] Charlwood JD, Graves PM (1987). The effect of permethrin-impregnated bednets on a population of *Anopheles farauti* in coastal Papua New Guinea. Med Vet Entomol.

[35] Govella NJ, Okumu FO, Killeen GF (2010). Insecticide-treated nets can reduce malaria transmission by mosquitoes which feed outdoors. Am J Trop Med Hyg.

[36] Chiang GL, Loong KP, Chan ST, Eng KL, Yap HH (1991). Capture-recapture studies with *Anopheles maculatus* Theobald (Diptera: Culicidae) the vector of malaria in peninsular Malaysia. Southeast Asian J Trop Med Public Health.

[37] Hii JL (1985). Evidence for the existence of genetic variability in the tendency of *Anopheles balabacensis* to rest in houses and to bite man. Southeast Asian J Trop Med Public Health.

[38] Hii JL, Chew M, Sang VY, Munstermann LE, Tan SG, Panyim S (1991). Population genetic analysis of host seeking and resting behaviors in the malaria vector, *Anopheles balabacensis* (Diptera: Culicidae). J Med Entomol.

[39] Russell TL, Govella NJ, Azizi S, Drakeley CJ, Kachur SP, Killeen GF (2011). Increased proportions of outdoor feeding among residual malaria vector populations following increased use of insecticide-treated nets in rural Tanzania. Malar J.

[40] Moiroux N, Gomez MB, Pennetier C, Elanga E, Djènontin A, Chandre F (2012). Changes in *Anopheles funestus* biting behavior following universal coverage of long-lasting insecticidal nets in Benin. J Infect Dis.

[41] Sougoufara S, Diedhiou S, Doucoure S, Diagne N, Sembene P, Harry M (2014). Biting by *Anopheles funestus* in broad daylight after use of long-lasting insecticidal nets: a new challenge to malaria elimination. Malar J.

[42] Reddy M, Overgaard H, Abaga S, Reddy V, Caccone A, Kiszewski A (2011). Outdoor host seeking behaviour of *Anopheles gambiae* mosquitoes following initiation of malaria vector control on Bioko Island, Equatorial Guinea. Malar J..

[43] Govella N, Chaki P, Killeen G (2013). Entomological surveillance of behavioural resilience and resistance in residual malaria vector populations. Malar J.

